# Could CA 19-9 be a useful biomarker in the diagnosis, prognosis, and prediction of adequate relief in lower urinary tract obstructions?

**DOI:** 10.1590/0100-6991e-20223304-en

**Published:** 2022-08-17

**Authors:** MARCIA EMILIA FRANCISCO SHIDA, HUMBERTO DELLÊ, MARCIA SILVA QUEIROZ

**Affiliations:** 1 - Universidade Nove de Julho, Pós-graduação stricto sensu em Medicina - São Paulo - SP - Brasil; 2 - Universidade Federal de São Paulo, Disciplina de cirurgia pediátrica, Urologia - São Paulo - SP - Brasil

**Keywords:** CA 19-9 Antigen, Lipocalin-2, Urinary Bladder Neck Obstruction, Antígeno CA 19-9, Lipocalina-2, Obstrução do Colo da Bexiga Urinária

## Abstract

**Introduction:**

posterior urethral valves represent an important cause of childhood chronic kidney disease. The identification of biomarkers that indicate early kidney damage and even adequate clearance could reduce how many patients head towards kidney failure.

**Objective:**

this study evaluated how this easy-analysis biomarker (CA 19-9) could help identifying potential renal damage and adequate clearance in obstructive uropathies.

**Methods:**

46 female Wistar rats were divided into 5 groups, with different patterns of partial urinary tract obstruction: group control; group OIV: infravesical obstruction; group OIVd: infravesical obstruction with reversion, obstruction relief 7 postoperative days later; group OUu: unilateral ureteral obstruction; group OUb: bilateral ureteral obstruction. The CA 19-9s performance was compared to another biomarker: Ngal. Determination of basal CA 19-9 and Ngal in urine and blood and serum creatinine levels was performed in the rats prior to surgery (T0) and after 14 days (T1). Group OIVd underwent intermediate (Ti) collection before clearance.

**Results:**

the urinary concentration of CA 19-9 increased in groups OIV, OIVd and OUb; elevation at T1 and Ti, reached statistical significance compared to the T0 value (p<0,05). Changes in urinary CA 19-9 were more expressive in infravesical obstruction groups (AUC 0.81). Obstruction relief in group OIVd promoted significant urinary CA 19-9 reduction (p<0,05) in the final evaluation.

**Conclusions:**

CA 19-9 urinary concentration increased in partial urinary tract obstruction. Its best performance was in the bladder neck obstruction group, in which the elevation was detected early (6 days after infravesical obstruction) and the CA19-9 urinary concentration declined after clearance.

## RESUMO

**Introdução:**

a válvula de uretra posterior representa uma importante causa de doença renal crônica na infância. A identificação de biomarcadores que monitorem danos renais precoces e o sucesso da desobstrução do trato urinário podem reduzir o número de pacientes que evoluem para insuficiência renal.

**Objetivo:**

avaliar o desempenho do biomarcador antígeno carboidrato CA 19-9 nas obstruções parciais do trato urinário.

**Método:**

46 ratas Wistar foram divididas em 5 grupos: grupo controle; grupo OIV: obstrução infravesical; grupo OIVd: obstrução infravesical com alívio da obstrução após 7 dias; grupo OUu: obstrução ureteral unilateral; grupo OUb: obstrução ureteral bilateral. O desempenho do CA 19-9 foi comparado a outro biomarcador, a Ngal. A dosagem de CA 19-9 e Ngal na urina e no sangue, e os níveis de creatinina sérica foram avaliados nas ratas antes da cirurgia (T0) e após 14 dias (T1). O grupo OIVd foi submetido a uma coleta intermediária (Ti).

**Resultados:**

a concentração urinária de CA19-9 aumentou nos grupos OIV, OIVd e OUb; a elevação em T1 e Ti alcançou significância estatística em relação ao valor de T0 (p<0,05). As alterações no CA 19-9 urinário foram mais expressivas nos grupos de obstrução infravesical (AUC 0,81). O alívio da obstrução no grupo OIVd promoveu redução do CA 19-9 urinário (p<0,05).

**Conclusões:**

a concentração urinária de CA19-9 aumentou na obstrução parcial do trato urinário. Seu melhor desempenho foi no grupo de obstrução infravesical, no qual a elevação foi detectada precocemente (6 dias de pós-operatório) com queda após a retirada do fator obstrutivo.

## INTRODUCTION

Congenital lower urinary tract obstruction (LUTO) and neurogenic bladder resulting from neural tube defects are diseases that cause urinary flow obstruction and can progress to obstructive nephropathy (ON) and chronic kidney disease (CKD)^1^. The posterior urethral valve (PUV) is the main etiology of LUTO^2^.

The first ultrasound sign of LUTO is fetal bladder distention (megacystis), diagnosed from 11 weeks of intrauterine life. Other findings include bilateral ureterohydronephrosis, thickened bladder, and dilated posterior urethra, the latter two being strongly correlated with PUV, together forming the Keyhole Sign^3^. Certain abnormalities of the renal parenchyma identified on ultrasound suggest greater severity in LUTO, especially when associated with oligohydramnios, such as increased echogenicity, presence of cystic structures, and thinning^4^.

The main concerns with LUTO are related to the best timing of the surgical intervention, whether prenatal or postnatal, and the prediction of progression to CKD^5^. In this sense, biomarkers can assist in decision making and improve patients’ prognosis. The CA 19-9 carbohydrate antigen is a marker of easy collection and well-standardized laboratory dosage and has shown promise in the literature as a marker of severity in ureteral obstructions, mainly related to Ureteropelvic Junction Stenosis (UPJS)^6^. However, it is still poorly studied in infravesical obstructions.

This experimental model intervention study was designed to assess urinary and serum CA 19-9 values in high and low partial urinary obstructions, and their behavior after bladder outlet release. We compared CA 19-9 with a second marker, Lipocalin associated with neutrophil gelatinase (Ngal), related to kidney injury and used to monitor acute renal failure^7^.

## METHODS

The experimental protocols were approved by the Animal Ethics Committee (CEUA UNINOVE nº 6575031019) and performed according to institutional guidelines.

We divided 46 adult Wistar rats (230-270 g) into five different groups: Control group (nine animals) - opening and closing of the abdominal wall; PBO group (nine animals) - partial bladder outlet obstruction for 14 days; PBOr group (10 animals) - partial bladder outlet obstruction for seven days and then release, with euthanasia 14 days after the first surgery; PUOu group (nine animals) - partial, high, and unilateral right ureteral obstruction for 14 days; PUOb group (nine animals) - partial, high, and bilateral ureteral obstruction for 14 days.

The surgical procedures were as follows: after anesthesia of the animal, infravesical obstruction (PBO) was performed after inferior longitudinal median laparotomy and careful exposure of the bladder to the bladder neck, with visualization of the ureterovesical junction and urethra. A 5-0 nylon suture was passed posteriorly to the bladder, over a 20G intravenous catheter (1.1×32mm) and tied to the anterior surface of the infravesical region. Then, the catheter was removed, and we verified the partial occlusion of the bladder with its compression and voiding through the urethra.

For the PBOr group, the obstruction was performed as described above, but we kept a loop of the nylon suture used to promote the obstruction attached to the subcutaneous tissue to facilitate its identification. On the 7^th^ postoperative day, this nylon thread was removed by inferior laparotomy to relieve the bladder outlet obstruction.

Partial ureteral obstruction followed the model proposed by ULM and MILLER (1962) and modified by other authors^8^ ^-^ ^11^. After median laparotomy, we identified the kidney, ureter, and psoas muscle. Then, we enclosed the right ureter just below the inferior lumbar vein by simply suturing the psoas muscle with a 5-0 prolene suture over a 20G venous catheter, causing partial ureteral obstruction. The same procedure was adopted in the left ureter in animals submitted to bilateral obstruction.

We carried out the experiments according to the following protocol: Day 0: the animals were housed in metabolic cages for urine collection to measure CA 19-9 and Ngal; Day 1: the animals were anesthetized, a blood sample was collected for measurement of CA 19-9, Ngal, and creatinine, and the surgical procedures were performed according to the animal’s allocation group; Day 13: the animals were again housed in metabolic cages for urine collection for measurement of CA 19-9 and Ngal; Day 14: the animals were anesthetized for blood collection to measure CA 19-9, Ngal, and creatinine; then they underwent euthanasia and surgical removal of the entire urinary tract. After urinary tract excision, the kidneys were weighed (grams) and measured longitudinally (millimeters). The bladder was also weighed and measured from the neck to the dome (millimeters).

The animals in the PBOr group had extra steps. On Day 6, they were housed in metabolic cages for urine collection for measurement of CA 19-9 and Ngal; on Day 7, they were anesthetized and underwent laparotomy for relief of obstruction and blood collection for CA 19-9, Ngal, and creatinine.

Comparisons of serum and urinary markers were performed at baseline (time 0 or T0) and on day 14 (time 1 or T1), and an intermediate value (Ti), before (urine) and during (blood) relief of obstruction was performed for the PBOr group.

We performed an immunohistochemical study with a CA 19-9 primary antibody (clone: 1116-NS-19-9, Dako Corporation AS, California, USA) in 29 kidneys and 13 bladders to demonstrate CA 19-9 expression and production site.

Statistical analysis was performed using the SPSS software package version 26.0 (IBM SPSS Inc., Chicago, IL) and GraphPad Prism 9.2.0 (California, USA). Data distribution and normality assessment were carried out using the Shapiro-Wilk test. Descriptive analysis of quantitative variables with parametric data was performed with mean and standard deviation and compared by the Student’s t test. When the evaluation of more than two samples was included, we used the ANOVA test with Bonferroni’s post-test correction. Nonparametric data were described as median and interquartile range and treated with the Mann Whitney test when comparing two groups or the Kruskal-Wallis test when analyzing more than two groups, with Dunnett’s post-test. The Wilcoxon test was used to determine the difference between pre and postoperative parameters. We applied the Receptor Operation Characteristics (ROC) curves for evaluating the diagnostic properties of the biomarkers in the studied groups in which there was significance in the elevation and/or fall of the levels within the same group. The cut-off value was determined using the Younden index^12^. The area under the curve (AUC) was considered satisfactory above 0.70; p-values less than 0.05 (p<0.05) were considered significant.

The number of animals needed for each group was calculated using the G-power 3.1.9ª program (Statistical Power Analyzes for Windows and Mac, http://www.gpower.hhu.de/), and the ANOVA statistical method, for an interval of 80% confidence and 5% alpha error.

## RESULTS

In the macroscopic evaluation of the urinary tract, we considered the longitudinal size of the kidney, the distance between the bladder neck and bladder dome, the thickness of the renal parenchyma and detrusor, and the weight of kidneys and bladder (Table 1). 

**Table 1:** Mean values of the gross anatomy of the obstructed groups compared to the measurements found in the control group.

Groups	Longitudinal size (mm)			weight (g)			Parenchyma (mm)		Detrusor (mm)
	RD	RE	B	RD	RE	B	RD	RE	B
Control	16.2±1.3	15.5±1.7	9.7±2.1	1.1±0.1	1.1±0.1	1.1±0.1	4.2±1.0	3.8±0.8	0.8±0.3
PBO	17.5±3.2	17.5±2.1	18.1±7.2*	1.2±0.2	1.1±0.1	0.8±0.7*	4.7±1.1	3.2±0.5	1.4±1.6
PBOr	17.8±2.2	17.2±1.8	14.1±3.6*	1.1±0.8	1.1±0.1	0.3±0.2*	4.2±1.1	4.8±1.3	0.9±0.5
PUOu	19.3±2.9*	-	-	1.3±0.1*	-	-	5.1±1.5	-	-
PUOb	18.5±2.2*	18.6±2.2*	-	1.1±0.1	1.1±0.2*	-	4.3±1.0	4.0±0.6	-

Legend: PBO: bladder outlet obstruction; PBOr: bladder outlet obstruction followed by release; PUOu: unilateral ureteral obstruction; PUOb: bilateral ureteral obstruction; RK: right kidney; LK: left kidney; B: bladder; g: grams; mm: millimeters; *p<0.05.

In the PBO group, at the time of laparotomy for euthanasia, we observed dilation of the entire urinary tract, but the most evident changes in relation to the control group were bladder size (p<0.05) and weight (p<0.05). In the PBOr group, even with a shorter time of bladder outlet obstruction (seven days) followed by release, bladder size and weight were significantly higher when compared with the control group (p<0.05 for both). In the PUOu group, the weight and size of the right kidney were significantly greater than in the control group (p<0.05), while in the PUOb group, renal size was greater than in the control on both on the right and on the left (p<0.05), and the weight was significantly greater on the left (p<0.05).

**Figure 1:**
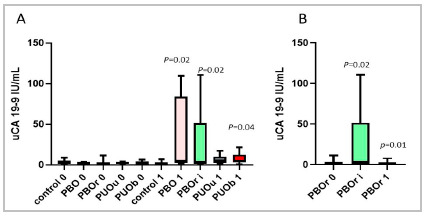
(A) Comparison of urinary concentrations of CA 19-9, initial (0) and after the surgical procedure (1 and i) in the groups: Control, Infravesical Obstruction (PBO) and Infravesical Obstruction followed by release (PBOr) analyzed with Wilcoxon test. The Unilateral Ureteral Obstruction (PUOu) and Bilateral Ureteral Obstruction (PUOb) groups were evaluated with the t test for paired samples. (B) Comparison of initial (0), intermediate (i) and after clearance (1) urinary CA 19-9 concentrations in the PBOr group using the Wilcoxon test.

**Figure 2:**
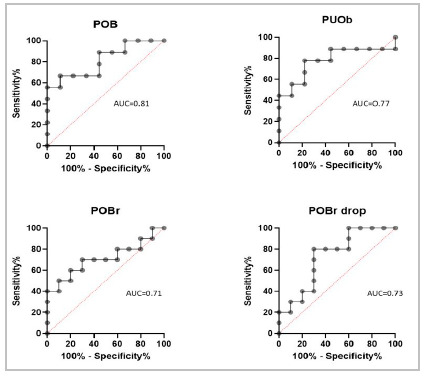
ROC curves determined by the increase in urinary CA 19-9 concentration after obstruction in the groups: PBO [AUC 0.81 (95% confidence interval 0.61-1) and cut-off value of 2.67 IU/ml]; PUOb [AUC of 0.77 (95% CI 0.54-1) and cut-off 3.68 IU/mL]; PBOr [AUC 0.71 (95% CI 0.47-0.94) and cut-off 2.79 IU/mL]. In the PBOr group, the ROC curve demonstrates the decrease in urinary CA 19-9 levels after release [AUC of 0.73 (95% CI 0.50-0.95) and cut-off 2.53 IU /mL].

Figure 1A shows the distribution of Urinary CA 19-9 (uCA 19-9) values for all groups. We observed an increase in uCA 19-9 after obstruction in all groups, with statistical significance in the increase in uCA 19-9 between T0 and T1 in the PBO and PUOb groups (p<0.05). Figure 2 shows the ROC curves of these groups.

In the PBOr group, there was a significant increase in uCA 19-9 between T0 and Ti (p<0.05), and after removal of the urinary tract obstruction, we observed a decrease between Ti and T1 (p<0.05) (Figure 1B) . Figure 2 shows the ROC curve for the drop in uCA 19-9 after release.

**Figure 3:**
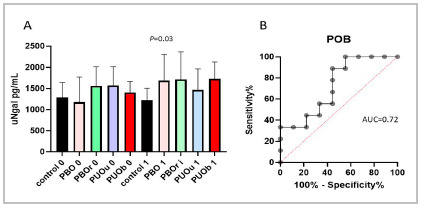
(A) Comparison of urinary Ngal concentrations, initial (0) and after the surgical procedure (1 and i), in each group (statistical method: t test for paired samples). (B) ROC curve determined by the elevation of urinary Ngal concentration in the PBO group [AUC 0.72 (95% CI 0.48-0.96) and cut-off 1101.68 pg/mL].

We compared the values of the urinary marker Ngal (uNgal) between the animals of the same group at the times already described. We observed an increase in uNgal in the PBO, PBOr, and PUOb groups, with statistical significance only in the PBO group (p<0.05). There was also a drop in this marker after release in the PBOr group, though not significant (Figure 3).

There were no significant changes in serum levels of CA 19-9 and Ngal. There was also no significant increase in serum creatinine in the groups submitted to urinary tract obstruction.

**Figure 4:**
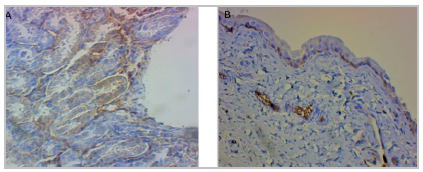
Immunohistochemistry of renal tubule and bladder. (A) CA 9-9 expression (brown color) in renal tubular cells (100X magnification) and (B) in the bladder lamina propria (40X magnification).

As for immunoexpression of CA 19-9, kidney positivity was 62.5% in the PUOb group, 83.3% in the control group, 87.5% in the PBO group, and 100% in the PBOr and PUOu groups. Bladder positivity was 50% in the PBOr group, 75% in the PUOb group, and 100% in the control and PBO groups (Figure 4).

## DISCUSSION

Experimental models of urinary tract obstruction (UTO) have provided a better understanding of the pathogenesis of obstructive nephropathy, as they allow the control of time, severity of renal lesions, and uniformity in structural changes, without the interference of confounding factors such as comorbidities or toxins, in addition to enabling the removal of the initial obstruction cause at different time intervals^13^ .

We found evidence in the urinary tract of macroscopic alterations secondary to obstruction to the flow of urine, characterized by bladder distention and ureterohydronephrosis, in addition to greater bladder size and weight, in animals in the PBO and PBOr groups, compared with the control group. The renal longitudinal size and the weight of the rats with ureteral obstruction were also significantly higher in relation to the control group. Thus, the effectiveness of the experimental models used was demonstrated. Similar parameters have been used in the literature to describe the efficiency of experimental bladder neck obstruction in rats^14^.

A better understanding of the mechanism of kidney injury in OTU indicates that biomarkers released by tubular epithelial elongation can provide valuable information about obstructive injury during childhood^15^ ^,^ ^16^. Through a review of the literature on OTU markers, we evidenced the immunohistochemical expression of the biomarker CA 19-9, originally a marker for neoplasms, in the cytoplasm of renal tubular cells, and its serum and/or urinary elevation has been described in patients with ureteropelvic obstruction (UPO)^17^ ^-^ ^19^ without associated malignancies.

Few studies have investigated the possible correlation between CA 19-9 and LUTO. One of them measured levels of Urinary CA 19-9 in pregnant women with fetuses with PUV and identified a significant increase compared with control pregnant women, which was related to a greater anteroposterior diameter of the fetal renal pelvis^20^.

In this study, the increase in urinary CA 19-9 values in the groups submitted to bladder outlet obstruction was more expressive when compared with ureteral obstructions, as can be seen in Figure 1 .

ROC curves indicated effectiveness of raising uCA 19-9. in PBO, PBOr, and PUOb groups, the AUC being 0.81, 0.71 and 0.77, respectively, and 0.73 in the PBOr group for post-release decrease. The value of uCA 19-9 in the PBOr group was significant and effective in the initial elevation, after six days of obstruction, proving to be important as a marker of precocity. In this same group, there was a decline in the urinary concentration of CA 19-9 after the obstruction was relieved, which may be important for the clinical and/or postoperative follow-up of LUTO patients.

The unilateral UPJS simulation (PUOu group), despite the increase in CA 19-9 values, did not present a statistically significant difference. Thus, it can be considered that bilateral obstruction, being more severe, causes a greater increase in urinary CA 19-9.

In the immunohistochemical analysis of the tissues of the urinary tract, we identified CA 19-9 in the bladders of control rats and of the obstructed groups. Thus, if we consider the bladder as a probable site of CA 19-9 production, in addition to the already known renal tubules and renal pelvis mucosa, we can explain the good sensitivity of this marker in bladder obstructions observed in this study .

As for uNgal as a comparative marker, we observed its increase after the urinary tract obstruction procedure, with significance only in the PBO group. The decrease in uNgal after release in the PBOr group was also not significant. The urinary concentrations of CA 19-9 increased significantly in three of the four obstructed groups and its decrease was also expressive with release, showing a better performance when compared with uNgal.

Serum measurements of markers present conflicting results in the literature, as serum CA 19-9 has been described as significantly elevated in OUT. However, it was not altered in the blood of patients with hydronephrosis in several studies^21^ ^,^ ^22^. We did not identify differences in the blood values of CA 19-9 and Ngal, before and after obstruction, between the different groups and in the comparison with the control group.

## CONCLUSIONS

The performance of urinary CA 19-9 as a marker was superior in urethral and bilateral ureteral obstructions. Urinary elevation of CA 19-9 was early, with six days of bladder outlet obstruction. There was a decline in the urinary concentration of CA 19-9 after clearance, which may be important for the clinical and/or postoperative follow-up of LUTOs.

The ease of use of uCA 19-9 and the results observed in this work indicate the importance of prospective studies in humans in the investigation of this biomarker as an auxiliary tool in decision making for expectant management or for early surgical intervention in OTUs. It could also contribute to the definition of the success or failure of obstruction relief or as a criterion in the indication of fetal interventions in the presence of congenital OTU.
